# Immunomodulation of RA Patients' PBMC with a Multiepitope Peptide Derived from Citrullinated Autoantigens

**DOI:** 10.1155/2017/3916519

**Published:** 2017-06-21

**Authors:** Smadar Gertel, Gidon Karmon, Sivan Vainer, Ora Shovman, Martin Cornillet, Guy Serre, Yehuda Shoenfeld, Howard Amital

**Affiliations:** ^1^Zabludowicz Center for Autoimmune Diseases, Sheba Medical Center, Tel-Hashomer 5262100, Ramat-Gan, Israel; ^2^Sackler Faculty of Medicine, Tel-Aviv University 6997801, Tel-Aviv, Israel; ^3^Department of Internal Medicine B, Sheba Medical Center 5262100, Tel Hashomer, Israel; ^4^Unité Différenciation Epithéliale et Autoimmunité Rhumatoïde, Université de Toulouse-INSERM, Toulouse, France; ^5^Laboratoire de Biologie Cellulaire et Cytologie, CHU de Toulouse, Toulouse, France; ^6^Incumbent of the Laura Schwarz-Kipp Chair for Research of Autoimmune Diseases, Tel Aviv University 6997801, Tel Aviv, Israel

## Abstract

Citrullinated peptides are used for measuring anticitrullinated protein antibodies (ACPA) in rheumatoid arthritis (RA). Accumulation of citrullinated proteins in the inflamed synovium suggests that they may be good targets for inducing peripheral tolerance. In view of the multiplicity of citrullinated autoantigens described as ACPA targets, we generated a multiepitope citrullinated peptide (Cit-ME) from the sequences of major citrullinated autoantigens: filaggrin, *β*-fibrinogen, vimentin, and collagen type II. We assessed the ability of Cit-ME or the citrullinated *β*60-74 fibrinogen peptide (*β*60-74-Fib-Cit) which bears immunodominant citrullinated epitopes (i) to modify cytokine gene expression and (ii) to modulate Treg and Th17 subsets in PBMC derived from newly diagnosed untreated RA patients. RA patient's PBMC incubated with Cit-ME or *β*60-74-Fib-Cit, showed upregulation of TGF-*β* expression (16% and 8%, resp.), and increased CD4^+^Foxp3^+^ Treg (22% and 19%, resp.). Both peptides were shown to downregulate the TNF-*α* and IL-1*β* expression; in addition, Cit-ME reduced CD3^+^IL17^+^ T cells. We showed that citrullinated peptides can modulate the expression of anti- and proinflammatory cytokines in PBMC from RA patients as well as the proportions of Treg and Th17 cells. These results indicate that citrullinated peptides could be active in vivo and therefore might be used as immunoregulatory agents in RA patients.

## 1. Introduction

Rheumatoid arthritis (RA) is an autoimmune disease estimated to affect nearly 1% of the adult population worldwide. It is characterized by progressive synovial inflammation resulting in irreversible joint destruction [1]. Many of the RA-specific autoantibodies are generated against citrullinated antigens and termed anticitrullinated protein antibodies (ACPA). These autoantibodies present a high diagnostic and prognostic value [2–4]. The targets for ACPA are citrullinated peptides, that is, peptides that underwent posttranslation conversion of arginine to citrulline residues catalyzed by peptidylarginine deiminases (PAD). Currently, citrullinated peptides serve merely as immunosorbents in the ACPA diagnostic assays. The four citrullinated proteins, filaggrin, fibrinogen, vimentin, and collagen type II, have been described as the major autoantigens in RA [5–7].

The pathogenesis of RA appears to be based on inflammatory responses mediated by self-reactive T and B cells reacting against citrullinated proteins. In autoimmune conditions, T cells reactive to self-antigens escape elimination in the thymus and are activated in the periphery where they can damage specific organs.

PAD isoforms are expressed in thymic epithelial cells; however, it is not clear whether a negative selection of citrulline-specific T cells occurs and whether those T cell clones manage to escape into the periphery [8]. In addition, the presence of ACPA is associated with RA susceptibility alleles such as HLA-DRB1, HLA-DRB4, and HLA-DRB10 genes [9, 10]. These alleles encode for a specific motif in the peptide-binding pocket of the human leucocyte antigen (HLA) bearing the so-called shared epitope (SE) [11]. Citrullination may enhance the immunogenicity of “altered-self” peptides through increased binding affinity to SE-containing HLA-DRB presented on MHC class II molecules. This may result in loss of tolerance to the related citrullinated antigens, mediated by activation of citrulline-specific-autoreactive T and B cells, leading to production of ACPA, then formation of immune complexes in the synovial tissue, and induction of the proinflammatory cytokine secretion by macrophages, all leading to synovial inflammation [9, 12–14].

The inflamed joints in RA contain a large number of T cells, the majority of which are CD4^+^ T cells [15]. The autoantigen that causes stimulation and expansion of specific T cell subsets should be present in the joint and presented by the HLA-DR alleles associated with RA. The fact that inflamed joints of RA patients contain large amounts of citrullinated proteins implies that they may be possible targets for tolerance induction [16]. Restoring self-tolerance without causing immunosuppression remains a major challenge.

In view of the multiplicity of citrullinated proteins described as target autoantigens for ACPA, we generated a multiepitope citrullinated peptide (Cit-ME) derived from the sequence of the major prevalent autoantigenic citrullinated proteins, namely, filaggrin, *β*-fibrinogen, vimentin, and collagen type II. Previously, we reported that the Cit-ME peptide ameliorates adjuvant-induced arthritis (AIA) in rats via induction of regulatory T cells and downregulation of Th17 cells [17, 18].

The current research analyzed the capacity of the same citrullinated multiepitope peptide, Cit-ME and of an additional single citrullinated fibrinogen peptide bearing immunodominant epitopes, to modify cytokine expression and modulate T cell subsets in PBMC from patients with RA.

## 2. Materials and Methods

### 2.1. Human Subjects and Sample Procurement

A total of 30 patients with RA (17 females and 13 males) were included in the study. All patients fulfilled the 2010 American College of Rheumatology (ACR) criteria for RA. The study protocol was approved by the Medical Ethics Review Board of Sheba Medical Center. All participants signed an informed consent form prior to the initiation of the study. We enrolled only newly diagnosed patients. Patients' age was 52.9 ± 13.7 (mean ± standard deviation) years, and 90% were ACPA positive (Table 1). Blood samples were collected before starting any therapy. The study was designed to test immunomodulation induced by citrullinated peptides and to assess their possible contribution to the pathogenesis of arthritis. The suggested controls were approved biological disease modifying antirheumatic drugs; therefore, the assays were conducted only on PBMC derived from patients with RA.

### 2.2. Detection of Antictrullinated Protein Antibodies (ACPA)

Sera from patients were tested for ACPA using the commercial ELISA QUANTA Lite® CCP3 IgG kit (Inova, USA), according to the manufacturer's instructions. Sera with results < 25 U/mL are defined as negative, and sera with results ≥ 25 U/mL, as positive.

### 2.3. Synthetic Peptides and Biotherapeutic Agents

**Figure 1:**
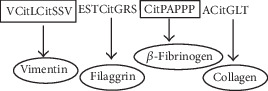
Scheme of Cit-ME peptide. Representative scheme of the multiepitope citrullinated peptide sequence delineated from the major autoantigens of anticitrullinated protein antibody (ACPA). The citrullinated residues are indicated (Cit).

The multiepitope citrullinated peptide Cit-ME (Figure 1) and its noncitrullinated counterpart containing arginine instead of citrulline residues, Non-Cit-ME, were obtained from GL Biochem (Shanghai, China). The citrullinated peptide *β*-Fib-Cit (*β*60-74Cit_60, 72, 74_) with the sequence CitPAPPPISGGGYCitACit derived from the *β* chain of human fibrinogen [19] and its noncitrullinated counterpart (*β*-Fib-NC) were obtained from NeoMPS (Strasbourg, France). The sequences of *β*-Fib-Cit and *β*-Fib-NC are also present in part in Cit-ME and Non-Cit-ME, respectively.

All peptides were added to cell culture medium at a concentration of 1.25 *μ*g/mL. This concentration was selected based on previous publications dealing with the capability of autoantigen peptides to induce proliferation at various concentrations of 2.5–10 ug/mL [20, 21]. We determined no proliferation at a concentration of 1.25 *μ*g/mL with different citrullinated peptides (data not shown); hence, this concentration was selected to confirm tolerizing regime. In the same way, infliximab or tocilizumab was added to cell culture medium at a concentration of 10 *μ*g/mL.

### 2.4. In Vitro Experiments

Peripheral blood mononuclear cells (PBMC) were isolated from heparinized venous blood using lymphoprep (Amersham). Cells were cultured in RPMI 1640 medium containing 10% bovine fetal serum supplemented with penicillin (100 U/mL), streptomycin (100 g/mL), 2 mmol/L L-glutamine, and 50 *μ*M 2-*β*-mercaptoethanol. Cells were incubated for 24–72 hours at 37°C with the experimental peptides, drugs, or medium alone as control.

### 2.5. Tregs and Th17 Staining

Tregs were identified by the analysis of CD4 and Foxp3 expression. Cells were labeled with anti-CD4 (eBioscience Inc., San Diego, CA, USA), then fixed and permeabilized with Foxp3 detection kit buffers, and stained with anti-Foxp3. In some experiments, anti-CD25 was used to identify CD4^+^CD25^+^Foxp3^+^ cells. Th17 cells were determined by addition of phorbol 12-myristate 13-acetate (PMA) (5 ng/mL) and ionomycin (1 *μ*M) (Sigma-Aldrich, Seelze, Germany) to the culture media for 6 hours. Monensin (eBioscience Inc.) was added in the last two hours of stimulation. For Th17 analysis, cells were stained with anti-CD3, followed by fixation and permeabilization (eBioscience Inc.), and then stained intracellularly with anti-IL-17. Cells were analyzed by a flow cytometer (FACScalibur, Becton Dickinson, Franklin Lakes, NJ, USA). Data analysis was conducted using FlowJo software (Tree Star, Ashland, OR, USA).

### 2.6. Real-Time PCR

RNA was isolated using the Total RNA Purification Plus Kit (Norgen Biotek, ON, CA) according to the manufacturer's instructions. For cDNA synthesis, 1 *μ*g total RNA was transcribed with cDNA transcription reagents using the High Capacity cDNA Reverse Transcription Kit (Invitrogen™, Carlsbad, CA, USA) according to the manufacturer's instructions. Gene expression was measured in real-time PCR performed on a StepOnePlus™ Real-Time PCR System (Applied Biosystems, Foster City, CA, USA) according to the manufacturer's instructions. Primer sequences (forward and reverse, resp.) were the following: human TGF-*β*5′-GACACCAACTATTGCTTCAG-3′ and 5′- CAGGCTCCAAATGTAGGG-3′, TNF-*α*5′-CCCAGGGACCTCTCTCTAATCA-3′ and 5′-GGTTTGCTACAACATGGGCTACA-3′, IL-1*β*5′-TGATGGCTTATTACAGTGGCAATG-3′ and 5′-GTAGTGGTGGTGGGAGATTCG-3′, IL-6 5′-CAATCTGGATTCAATGAGGAGAC-3′ and 5′-TGTTCCTCACTACTCAAATCT-3′, IL-8 5′-TGGCAGCCTTCCTGATTTCT-3′ and 5′- GGGTGGAAAGGTTTGGAGTATG-3′ and CCL-3 5′-CCGGTGTCATCTTCCTAACC-3′ and 5′-TTCTGGACCCACTCCTCACT-3′, and GAPDH 5′-GAAGGTGAAGGTCGGAGTC-3′ and 5′- GAAGATGGTGATGGGATTTC-3′. The expression level of a gene in a given sample was represented as 2^−ΔΔCt^ where ΔΔCT = [ΔCT_(experimental)_] − [ΔCT_(medium)_] and ΔCT = [CT_(experimental)_] − [CT_(housekeeping)_]. The GAPDH levels were used to normalize gene expression levels.

### 2.7. Statistical Analysis

Statistical comparisons were conducted using the two-tailed Mann Whitney *U* test and Student *t-*test (GraphPad/Prism version 5 software, San Diego, CA, USA).

## 3. Results

### 3.1. Low Doses of Citrullinated Peptides Change Gene Expression of Anti- and Proinflammatory Cytokines in Cultured PBMC from RA Patients

To detect whether the multiepitope citrullinated peptide Cit-ME and the individual citrullinated peptide *β*-Fib-Cit could induce tolerance through immunomodulation of immune cells in RA, we studied their in vitro effects on cytokine gene expression and T cell subsets of cultured PBMC obtained from RA patients.

The PBMC were cultured for 24 hours in the absence or presence of the citrullinated peptides and as controls their arginine counterparts, at a concentration of 1.25 *μ*g/mL or with infliximab at 10 *μ*g/mL. Thereafter, mRNA was isolated from the cells, and the level of TGF-*β*, TNF-*α*, and IL-1*β* gene expression was determined by real-time RT-PCR.

**Figure 2:**
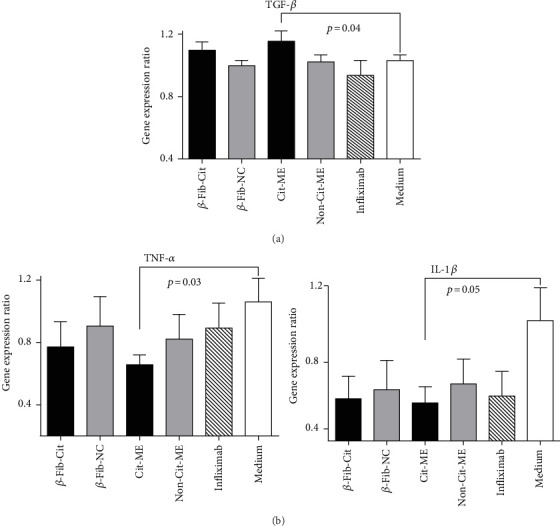
Cit-ME peptide upregulates TGF-*β* and downregulates TNF-*α* and IL-1*β* mRNA expression. PBMC of RA patients (*n* = 12) were cultured (5 × 10^5^ cells/well) for 24 hours in the presence of citrullinated peptides (1.25 *μ*g/mL). TGF-*β*, TNF-*α*, and IL-1*β* mRNA gene expression was determined by real-time RT-PCR. Results are presented as the mean ± SE percentage of gene expression.

As shown in Figure 2(a), Cit-ME peptide significantly upregulated the TGF-*β* gene expression level in the PBMC (*p* = 0.04) compared to incubation of cells with medium alone (normalization was performed to GAPDH and medium control was considered as 1). The peptide *β*-Fib-Cit seemed to also upregulate TGF-*β* gene expression, but this was not significant with the small number of patients studied. None of the noncitrullinated peptides produced any upregulation of the TGF-*β* gene expression compared to the medium. In contrast, incubation of cells with infliximab resulted in decreased TGF-*β* expression, compared to medium control.

Concomitantly, the Cit-ME peptide significantly downregulated the proinflammatory TNF-*α* and IL-1*β* expression levels as compared to the medium control (*p* = 0.03 and *p* = 0.05, resp.), as shown in Figure 2(b). The *β*-Fib-Cit peptide showed a tendency towards reducing TNF-*α* and IL-1*β* levels compared to the medium, but this remained nonsignificant. The noncitrullinated peptides were less potent in reducing TNF-*α* and IL-1*β* levels compared to their counterpart citrullinated peptides. Infliximab had no effect on the TNF-*α* level but reduced the mean IL-1*β* level compared to the medium control (*p* = 0.07).

### 3.2. The Cit-ME Peptide Downregulates IL-6, IL-8, and CCL3 mRNA Expression in Cultured PBMC from RA Patients

**Figure 3:**
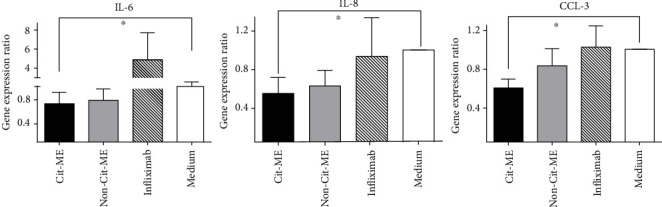
Cit-ME peptide downregulates IL-6, IL-8, and CCL-3 mRNA expression. PBMC of RA patients (*n* = 7–10) were cultured (5 × 10^5^ cells/well) for 24 hours in the presence of Cit-ME (1.25 *μ*g/mL). mRNA gene expression was determined by real-time RT-PCR. Results are presented as the mean ± SE percentage of gene expression, ⁣^∗^*p* < 0.02.

Since Cit-ME peptide immunomodulates the expression of essential inflammatory genes, it was of interest to find out whether it might alter the expression of additional related genes. We measured cytokine gene expression typically involved in innate immune responses, namely, IL-6, IL-8, and CCL-3.  Figure 3, represents cytokine gene expression levels (normalization was performed to GAPDH and medium control was considered as 1). In accordance with the expression pattern of the proinflammatory TNF-*α* and IL-1*β*, incubation of Cit-ME with PBMC of RA patients resulted in a significant downregulation in IL-6, IL-8, and CCL-3 gene expression as compared to incubation with medium alone (*p* < 0.02). However, the control peptide Non-Cit-ME could also downregulate significantly the IL-6 expression (*p* < 0.02) as compared to medium control. Whereas, the control peptide Non-Cit-ME or infiliximab could not downregulate significantly the IL-8 and CCL-3 expression as compared to medium control in cultured RA patients' PBMC.

### 3.3. The Cit-ME Peptide Affects Treg and Th17 Proportions in Cultured PBMC from RA Patients

**Figure 4:**
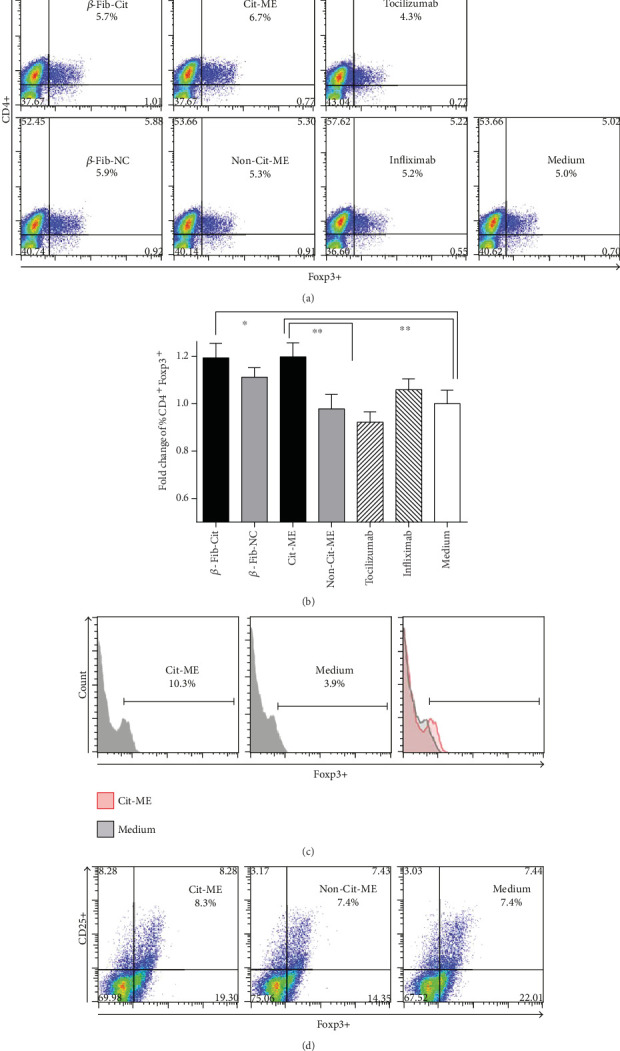
Cit-ME upregulates Treg in vitro. (a) Representative dot plot of CD4^+^Foxp3^+^ T cells from a RA patient. Positive staining is presented in the right upper quadrant of each graph with the percentage indicated. (b) The fold change of % CD4^+^Foxp3^+^ T cells determined by flow cytometry (*n* = 6–13). Data are presented as mean values (⁣^∗^*p* = 0.04, ⁣^∗∗^*p* = 0.02). (c) Histogram shows representative Foxp3 staining for one RA patient, gated for CD4^+^ T cells. (d) Representative dot plot of RA patient PBMC gated for CD4 and analyzed for CD25 and Foxp3 expression.

The effects of Cit-ME peptide on Treg were analyzed in cultured PBMC from RA patients. PBMC (2 × 10^6^) were cultured with Cit-ME, *β*-Fib-Cit, matched noncitrullinated peptides (Non-Cit-ME and *β*-Fib-NC), or medium alone. We also tested whether infliximab and tocilizumab could affect Treg proportion as well. Samples were analyzed by flow cytometry 72 hours after incubation, to measure the frequency of CD4^+^Foxp3^+^ T cells.  Figure 4(a) shows representative plot of ACPA^+^-RA patient PBMC stained with anti-CD4 and anti-Foxp3. As shown in Figure 4(b), Cit-ME significantly increased the fold change of % CD4^+^Foxp3^+^ T cells (*n* = 13, average of 1.22 ± 0.06) compared to the Non-Cit-ME (*n* = 13, average of 1.0 ± 0.07) or medium (*n* = 12, considered as 1) (*p* = 0.02), respectively. In addition, incubation with the *β*-Fib-Cit peptide resulted in significant increase in fold change of % CD4^+^Foxp3^+^ T cells (*n* = 6, average of 1.19 ± 0.07) as compared to medium alone, but as compared to *β*-Fib-NC (*n* = 6, average of 1.1 ± 0.04), variation was nonsignificant. Neither infliximab (*n* = 10, average of 1.07 ± 0.05) nor tocilizumab (*n* = 11, average of 0.99 ± 0.05) significantly altered the fold change of % CD4^+^Foxp3^+^ T cells compared to the medium control. Higher expression of Foxp3 on gated CD4 T cells in PBMC of representative RA patient that were incubated with Cit-ME compared to medium alone is shown (Figure 4(c)). This was true also for CD4^+^CD25^+^Foxp3^+^ cells that were elevated in RA patients' PBMC incubated with Cit-ME (8.3%) compared to Non-Cit-ME or medium control (7.4%) (Figure 4(d)).

**Figure 5:**
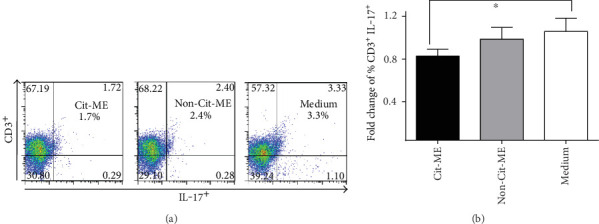
Cit-ME downregulated Th17 cells in vitro. (a) Representative plots of CD3 and IL-17 staining. Positive staining is presented in the right upper quadrant of each plot with the percentage indicated. (b) Fold change of % CD3^+^ IL-17^+^ T cells (*n* = 11). Data are presented as mean values (⁣^∗^*p* = 0.05).

Figure 5(a) shows representative plot of ACPA^+^-RA patient PBMC stained with anti-CD3 and anti-IL-17. As shown in Figure 5(b), after incubation with the Cit-ME peptide, significant reduction in the fold change of % CD3^+^IL-17^+^ T cells was detected (*n* = 9, average of 0.77 ± 0.08) compared to the medium control (*n* = 9, considered as 1) (*p* = 0.05). Incubation with the Non-Cit-ME peptide resulted in reduction in the fold change of % CD3^+^IL-17^+^ T cells (*n* = 7, average of 0.9 ± 0.1) as compared to the medium control alone, but the variations were nonsignificant.

## 4. Discussion

We have shown that Cit-ME, a multiepitope citrullinated peptide, has the capacity to immunomodulate pro- and anti-inflammatory gene expression in RA patients' PBMC. In vitro Cit-ME induced Treg and decreased the number of Th17 cells. In a previous study, we demonstrated that the tolerogenic Cit-ME peptide ameliorated manifestations of arthritis in a rat model of adjuvant-induced arthritis (AIA). The mechanisms leading to suppression of arthritis by Cit-ME peptide in vivo were also mediated by altering the Treg/Th17 balance and by increasing T cell apoptosis [17].

ACPA are known to appear years prior to arthritis, and citrullinated proteins are assumed to be the original target antigens in RA and perhaps the immunogens [22]. Hence, they might be the key to restoring immune tolerance and prevent impending disease. The approach we present here is based on inducing peripheral tolerance using a synthetic peptide derived from sequences of multiple prevalent citrullinated autoantigens. Indeed, the citrullinated *β*-fibrinogen peptide was also able to immunomodulate immune cells of RA patients even if to a limited extent compared to the Cit-ME peptide.

Stimulation of PBMC from 51 RA patients with the 5 different citrullinated fibrinogen peptides (50 *μ*g/mL) demonstrated that they significantly suppressed the T cell proliferative responses and did not change the production levels of IFN-*γ* and IL-17 [23]. These findings clearly indicated that the citrullinated fibrinogen peptides did not induce an immunogenic response but rather generated a tolerogenic phenotype.

Moreover, in a clinical trial conducted on ACPA^+^-RA patients carrying HLA-DRB1 SE allele's, immunotherapy of autologous dendritic cells loaded with four different citrullinated peptide antigens improved a patient's clinical state. The observed effects were associated with reduction of effector T cells and reduced proinflammatory cytokines in the serum with an increased ratio of regulatory to effector T cells [24].

On the other hand, studies that evaluated immune responses towards different citrullinated proteins, such as citrullinated vimentin and citrullinated aggrecan in RA patients, demonstrated a certain degree of immune cell stimulation directed to these citrullinated proteins [25, 26].

The dose of autoantigen used is a critical parameter, vimentin and aggrecan citrullinated peptides could induce certain proliferative response in T cells from RA patients at a concentation range of 2.5–10 *μ*g/mL [20]. Also, 5 *μ*g/mL of TSH peptides induce T cell response in vitro in PBMC derived from Graves' disease patients [21]. Here, citrullinated peptides were administered at a concentration of 1.25 *μ*g/mL chosen on a basis of their potential ability of tolerance induction. Possibly, such an approach induces TCR signals that are insufficient to fully activate T cells and, therefore, lead to functional T cell suppression by either energy or clonal deletion [27].

It was shown that RA patients have significantly higher frequencies of Cit-specific T cells and that a greater proportion of these cells displays a Th1 memory phenotype [28]. Thus, the administration of low-dose citrullinated peptides might assist to reverse the pathogenic autoreactive immune response by inducing immunomodulation.

We previously reported that Cit-ME effectively inhibited the course of AIA in Lewis rats. These therapeutic effects were associated with increased Treg and T cell apoptosis and fewer Th17 cells in the treated rats [17].

Here, the in vitro effect of Cit-ME on CD4^+^Foxp3^+^ and CD3^+^IL-17^+^ T cells was assessed; Cit-ME and *β*-Fib-Cit upregulated the CD4^+^Foxp3^+^ T cells. Further, characterizing the suppressive capability of such population is required. Phenotypical analysis of Treg into subgroups of activated Treg (CD45RA^−^Foxp3^high^), resting Treg (CD45RA^+^Foxp3low), which are both suppressive in vitro, and cytokine-secreting nonsuppressive non-Treg (CD45RA^−^FoxP3^low^) as shown could be more informative [29].

For instance, heat shock protein 60, altered peptide ligand (APL1), an additional autoantigen possibly involved in the pathogenesis of RA, was used for the induction of peripheral tolerance. Stimulation of PBMC from RA patients by APL1 increased the proportions of Treg [30], induced apoptosis of activated CD4^+^ T cells, presumably through a Treg-dependent mechanism [31], and increased Treg suppression against APL1 responding effector T cells, at the same time decreased IL-17 that is produced by activated T cells [32].

Similarly, in systemic lupus erythematosus (SLE), abnormal apoptosis may lead to extracellular release of nucleosomes, which include various histone proteins that are recognized as neoepitopes [33, 34]. Supplementing PBMC cultures of lupus patients with histone nucleosomal peptides induced an increase of Treg and suppressed IFN-*α* gene expression. Each of the histone peptidic epitopes also inhibited the pathogenic IgG autoantibody production in the lupus patients' PBMC. Combinations of these epitopes were even more efficient in suppressing pathogenic autoantibody production [35]. Accordingly, administration of low-dose histone-nucleosomal peptides induced tolerance in humans [36].

In our study, the anti-inflammatory response phenomenon exerted by the citrullinated peptides was compared to approved biological medications in RA (infliximab and tocilizumab). Addition of the anti-TNF-*α* agent infliximab did not affect significantly TGF-*β*, TNF-*α*, IL-8, and CCL-3 gene expression, while IL-1*β* mRNA was significantly decreased and IL-6 mRNA was elevated compared to the medium control. The observed effects on lymphocytes following stimulation with infliximab can indicate on the potential drug induce target genes in naïve RA patients' PBMC. We could not detect changes in Treg population following the addition of an anti-TNF-*α* (infliximab) or an anti-IL6 receptor agent (tocilizumab). However, anti-TNF therapy in RA patients was shown to have the capacity to generate newly differentiated population of Treg cells [37].

Study limitations are the following: first, the immunomodulatory effects of the citrullinated peptides were compared to the noncitrullinated matched peptides. Although the latter peptides could not promote equivalent immunomodulation, we cannot exclude that such control peptides may have a biological effect since they possess sequence homologies to known autoantigens and might evoke certain immune responses. Scrambled peptides should be considered as a better control for Cit-ME and *β*60-74-Fib-Cit activity.

Second, the citrullinated peptides administered in our study could immunomodulate cytokine expression also in ACPA seronegative patients (*n* = 3), indicating that although the ACPA titers measured were below the cut-off of the commercial ELISA kit for ACPA detection, the citrullinated peptides could nevertheless stimulate the PBMC and alter the mRNA profile of the cytokines. Further studies are required to determine the mechanistic effects of citrullinated peptides in ACPA seronegative RA patients.

## 5. Conclusion

In the present study, we have shown that a multiepitope citrullinated peptide could be an attractive target for therapeutic intervention in RA patients. This peptide may stimulate the expression of anti-inflammatory cytokines and downregulate proinflammatory cytokines in T cells. These modifications may shift the Treg/Th17 imbalance and restore homeostasis in arthritis.

## Figures and Tables

**Table 1:** Characterization of rheumatoid arthritis patients analyzed in the in vitro assays.

Patient number	Gender	Age	ACPA status	ACPA value
1	Female	35	Negative	12
2	Female	58	Moderate	49
3	Female	36	Moderate	48
4	Female	32	Weakly positive	26
5	Male	50	Highly positive	138
6	Female	38	Positive	78
7	Female	51	Negative	4
8	Male	49	Highly positive	242
9	Female	50	Positive	55
10	Female	50	Highly positive	258
11	Male	35	Highly positive	195
12	Female	68	Positive	61
13	Female	33	Highly positive	128
14	Male	53	Highly positive	211
15	Female	64	Highly positive	600
16	Male	60	Negative	3
17	Female	45	Positive	41
18	Male	70	Highly positive	210
19	Male	67	Highly positive	3700
20	Male	57	Highly positive	191
21	Female	55	Highly positive	171
22	Female	48	Moderate	57
23	Male	22	Highly positive	121
24	Female	65	Highly positive	227
25	Male	58	Negative	6
26	Female	75	Highly positive	210
27	Male	72	Highly positive	210
28	Male	63	Highly positive	210
29	Male	60	Highly positive	210
30	Female	70	Highly positive	150
